# Molecular Targeted Therapies for the Treatment of Leptomeningeal Carcinomatosis: Current Evidence and Future Directions

**DOI:** 10.3390/ijms17071074

**Published:** 2016-07-05

**Authors:** Dae-Won Lee, Kyung-Hun Lee, Jin Wook Kim, Bhumsuk Keam

**Affiliations:** 1 Department of Internal Medicine, Seoul National University Hospital, Seoul 03080, Korea; silver2sky@hanmail.net (D.-W.L.); drleekh@gmail.com (K.-H.L.); 2 Cancer Research Institute, Seoul National University College of Medicine, Seoul 03080, Korea; 3 Department of Neurosurgery, Seoul National University Hospital, Seoul 03080, Korea; wook616@hanmail.net

**Keywords:** leptomeningeal carcinomatosis, lung cancer, breast cancer, molecular targeted agent

## Abstract

Leptomeningeal carcinomatosis (LMC) is the multifocal seeding of cerebrospinal fluid and leptomeninges by malignant cells. The incidence of LMC is approximately 5% in patients with malignant tumors overall and the rate is increasing due to increasing survival time of cancer patients. Eradication of the disease is not yet possible, so the treatment goals of LMC are to improve neurologic symptoms and to prolong survival. A standard treatment for LMC has not been established due to low incidences of LMC, the rapidly progressing nature of the disease, heterogeneous populations with LMC, and a lack of randomized clinical trial results. Treatment options for LMC include intrathecal chemotherapy, systemic chemotherapy, and radiation therapy, but the prognoses remain poor with a median survival of <3 months. Recently, molecular targeted agents have been applied in the clinic and have shown groundbreaking results in specific patient groups epidermal growth factor receptor (*EGFR*)-targeted therapy or an anaplastic lymphoma kinase (*ALK*) inhibitor in lung cancer, human epidermal growth factor receptor 2 (*HER2*)-directed therapy in breast cancer, and CD20-targeted therapy in B cell lymphoma). Moreover, there are results indicating that the use of these agents under proper dose and administration routes can be effective for managing LMC. In this article, we review molecular targeted agents for managing LMC.

## 1. Introduction

Leptomeningeal carcinomatosis (LMC) is the multifocal seeding of leptomeninges by malignant cells. The incidence of LMC is approximately 5% of patients with malignant tumors and the rate of identification is increasing due to improvements in neuroimaging technologies and advances in cancer treatments [1,2,3]. Small cell lung cancer (SCLC) and melanoma exhibit the highest leptomeningeal spread rates. About 11% of SCLC patients develop LMC, and while meningeal involvement is clinically suspected in 10.6% of malignant melanoma patients, it is actually confirmed during autopsy in 52% of these cases [4,5]. However, most cases of LMC arise in breast cancer and non-small cell lung cancer (NSCLC) because of the high incidences of these cancers [3,6,7].

The prognosis of LMC remains very poor with a median overall survival of <3 months, but prognosis is influenced by the primary tumor type [3,7,8,9,10]. In a systemic review of clinical studies, patients with LMC from breast cancer show better survival rates (15.3 weeks) compared to those from lung cancer (8.7 weeks) [10]. Treatments are aimed at improving or stabilizing neurologic symptoms and prolonging survival. Radiation therapy, systemic chemotherapy, and intrathecal chemotherapy are primary treatments for LMC. Only six randomized clinical trials have been conducted for LMC and a standard treatment remains to be established [8,11,12,13,14,15]. Advances in molecular targeted agents have prolonged survival, especially in patients with lung cancer harboring epidermal growth factor receptor (*EGFR*) mutations or anaplastic lymphoma kinase (*ALK*) rearrangement, and in patients with breast cancer harboring human epidermal growth factor receptor 2 (*HER2)* amplification. The use of proper molecular targeted agents can prolong survival of patients with LMC relative to historical examples from pre-molecular targeted agents era [16,17,18,19]. In this article, we review recent results from the use of molecular targeted agents and discuss novel approaches to treating LMC.

## 2. Clinical Manifestation and Diagnosis

LMC usually occurs in patients with prolonged disease periods and disseminated systemic cancer. However, 5%–10% of LMC occurs in patients who show no evidence of systemic disease [20]. LMC presents with signs and symptoms of multifocal neuraxis disease. In a systemic review of 456 patients with LMC, common clinical manifestation included cranial nerve palsies (75%), headache (66%), cerebral disturbances (66%), spinal nerve symptom (60%), mental change (45%), limb weakness (44%) and difficult walking (33%) [21]. Nausea and vomiting also occurred for 20% of patients.

The gold standard for diagnosing LMC is cerebrospinal fluid (CSF) examination through lumbar puncture. While lumbar puncture is a relatively safe procedure, there can be severe complications such as cerebral herniation, meningitis, and bleeding in epidural or subdural spaces. Before performing a lumbar puncture, clinicians should be aware of any bulky intracranial diseases or bleeding diathesis. Initial CSF cytology is positive in 50% of cases and repeated spinal fluid analyses up to three times maximum yields up to 90% positive cytology [7,22]. Hence, repeated CSF cytology examination is needed for cytology-negative, clinically-suspicious cases. Elevated CSF opening pressure (found in 42%–70% of patients), high CSF white blood cell (WBC) counts (48%–64%), elevated CSF protein levels (59%–80%), and low CSF glucose levels (22%–58%) can support an LMC diagnosis in CSF cytology-negative patients [6,17,18,22,23]. There is also evidence that biochemical markers such as vascular endothelial growth factor (VEGF), CYFRA 21-1, neuron-specific enolase (NSE), and carcinoembryonic antigen (CEA) in the CSF can aid LMC diagnosis [24,25]. Detecting malignant cells in CSF by assessing circulating tumor cells have shown high sensitivity in LMC of breast cancer patients [26]. In addition, CSF-derived cell-free circulating tumor DNA has shown to complement the diagnosis of LMC [27]. However, use of such methods is limited due to poor sensitivity, poor specificity, and the lack of an accurate cutoff level. Clinicians should measure CSF opening pressure and obtain CSF cytology, cell counts, protein levels, and glucose levels in suspected LMC patients.

Magnetic resonance imaging (MRI) has become an important diagnostic tool for LMC following improvements in the quality of neuroimaging [6,28,29,30]. MRI is effective in LMC diagnosis for 83%–85% of solid tumor cases, but this sensitivity drops to 20%–50% in hematologic malignancies [6,28,31]. As observed through MRI, pial enhancement and nodularity are the most common features of LMC along with nodular disease, neural enhancement, and white matter changes [29]. MRI results that do not show LMC features are not sufficient to exclude an LMC diagnosis in clinically suspected cases. However, in patients with suspected disease, MRI alone is adequate for LMC diagnosis. MRI should be performed before lumbar puncture because meningeal irritation due to the puncture could yield false positive MRI results.

In suspicious patients, the LMC diagnosis could be made by CSF examination or MRI imaging. Recently, a metabolomic approach showed efficacy for LMC diagnosis [32] where the use of five metabolites from CSF resulted in diagnostic sensitivity and specificity of over 90%. Metabolomic approaches could augment current diagnostic modalities for LMC in the near future.

## 3. Treatment

Treatment goals for LMC are to improve neurologic symptoms and prolong survival. Because eradication of LMC is not yet possible, clinicians should carefully assess a patient’s overall clinical status to determine the degree or extent of treatment. The standard of treatment for LMC has not yet been established due to low incidence rates, the rapidly progressing nature of the disease, heterogeneous LMC populations, and a lack of sufficient randomized trial-based results. Treatment options for LMC include intrathecal chemotherapy, systemic chemotherapy and radiation therapy. However, the selection of these treatment modes is based on limited numbers of randomized clinical trials and most evidence on treatment effectiveness comes from non-randomized or observational studies.

### 3.1. Intrathecal and Systemic Chemotherapy

Achieving therapeutic dose levels of chemotherapeutic agents in the CSF space is challenging because of the blood-brain barrier (BBB) and the blood-CSF barrier [33,34]. BBB permeability is increased in patients with brain metastases through tumor perturbation and by the effect of antigen-rich sites within brain metastases [34,35]. High-dose methotrexate (MTX) and cytosine arabinoside (Ara-C) have historically been used as systemic chemotherapeutic agents to treat LMC, but results from systemic MTX and Ara-C use are minimal from lung cancer and breast cancer cases. These agents also show high toxicity and low efficacy for managing extracranial lesions. A pilot study of bevacizumab combined with etoposide and cisplatin in breast cancer patients with LMC showed promising efficacy and additional studies are needed to validate this finding [36].

Intrathecal chemotherapy was introduced in the 1970s to circumvent BBB and blood-CSF barrier issues. Chemotherapeutic agents can be delivered directly into the CSF space by lumbar puncture or through an intraventricular catheter. Intraventricular catheters, such as an Ommaya reservoir, are preferred because they are more comfortable for patients and are effective at delivering a uniform distribution of drug into the CSF space [37,38]. Distribution of the chemotherapeutic agent is dependent on CSF flow dynamics and a CSF flow scan should be performed before intrathecal chemotherapy [39].

Since the advent of the intrathecal chemotherapy, the most commonly used agents for intrathecal chemotherapy have been MTX, Ara-C, and thiotepa [8,12,13,14]. These drugs have shown efficacy as single agents or in combinations. MTX showed similar median survival rates (15.9 weeks vs. 14.1 weeks) to thiotepa in a study where 52 assessable patients were treated [12]. However, mucositis and neurologic complications were more common in patients who received MTX. Intrathecal sustained-release Ara-C was compared to intrathecal MTX in 61 LMC patients [14], but there was no significant difference in median survival between sustained-release Ara-C and MTX (105 days vs. 78 days, respectively; *p* = 0.15). However, median time to neurological progression favored sustained-release Ara-C over MTX (58 days vs. 30 days, *p* = 0.007). In a single-group, retrospective study a three-drug combination (intrathecal MTX, Ara-C, and hydrocortisone) showed more favorable effects than intrathecal MTX alone [40]. However, the combination of intrathecal MTX and Ara-C was not better than intrathecal MTX alone in a randomized clinical study [8]. Current evidence cannot support a preferable intrathecal chemotherapeutic agent from MTX, Ara-C, and thiotepa. Until more evidence is gathered, using any of the three agents is acceptable for managing LMC. Common complications from intrathecal chemotherapy include chemical aseptic meningitis, which occurs in >40% of patients [41]. Symptoms can be managed in an outpatient setting with oral antipyretics, antiemetics, and corticosteroids [20].

### 3.2. Radiotherapy and Surgery

Radiation therapy plays a critical role in brain metastases. However, whole brain radiotherapy did not improve survival in 125 lung cancer patients with leptomeningeal metastasis [42]. CSF fluid circulates from the ventricular system of the brain to the spinal cord and an extended radiation field is needed to treat LMC, which may result in significant radiation toxicity. Radiation therapy plays a limited role in managing LMC and is typically only used to correct CSF flow by focal mass or debulking mass adjustments that facilitate intrathecal chemotherapy efficacy [43]. Debulking surgery is required in cases where a large mass is present. Intraventricular catheters (e.g., Ommaya reservoirs) may be implanted in patients who are preparing for intrathecal chemotherapy. Ommaya reservoir placement is a safe procedure, and both frameless or frame-based techniques are safe and accurate [44].

## 4. Molecular-Targeted Agents in Leptomeningeal Carcinomatosis (LMC)

### 4.1. Lung Cancer Patients with Epidermal Growth Factor Receptor (EGFR) Mutations

NSCLC can be classified into several subtypes based on genetic profile. Mutations in the *EGFR* gene and rearrangement of the *ALK* gene are the two most studied NSCLC genetic profiles. *EGFR* mutations are found in 10%–15% of Caucasian NSCLC cases and 30%–40% of Asian NSCLC cases [45,46]. Identifying the mutation status of the *EGFR* gene is important because patients with *EGFR*-activating mutations can be effectively treated with *EGFR* tyrosine kinase inhibitor (TKI) [47,48]. *EGFR* mutations are also independent positive prognostic factors in NSCLC patients with brain metastases [49,50].

*EGFR* TKI is a treatment of choice in cases of NSCLC that also have *EGFR* mutations. Although there have been no randomized clinical trials, retrospective and historical data show that *EGFR* TKI may be a therapeutic option for LMC (Table 1). In a study by Liao, et al. [51]*,* *EGFR*-mutated patients who underwent *EGFR* TKI therapy for LMC showed longer overall survival compared to patients who did not (10.9 months vs. 2.3 months, *p* < 0.001). *EGFR* TKI can cross the BBB, but only at low levels of 1%–3% [52]. To achieve adequate therapeutic dosing with *EGFR* TKI in CSF, high-dose *EGFR* TKI has been tested in NSCLC with LMC. In a phase I study, high-dose gefitinib (750 or 1000 mg daily) resulted in neurologic symptom improvement in 57% of NSCLC patients who had shown prior response to *EGFR* TKI [53]. In addition, high-dose erlotinib (200 mg on alternate days, 300 mg on alternate days, 300 mg every 3 days, 450 mg every 3 days, or 600 mg every 4 days) proved to be effective and safe for managing LMC in patients who failed to respond to standard-dose *EGFR* TKI [54]. Both erlotinib and gefitinib showed efficacy in NSCLC with LMC. However, compared to gefitinib, erlotinib showed higher CSF concentration (28.7 vs. 3.7 ng/mL, *p* = 0.0008) and penetration levels (2.77% vs. 1.13%, *p* < 0.0001) [52]. In a retrospective study by Lee et al., erlotinib showed higher cytologic conversion rates compared to gefitinib in NSCLC with LMC (64.3% vs. 9.1%, *p* = 0.012) [55]. In *EGFR-*mutated NSCLC with LMC, erlotinib could be more effective than gefitinib, and high-dose *EGFR* TKI may be an appropriate option.

Afatinib, an irreversible second-generation *EGFR* TKI, is approved by the U.S. Food and Drug Administration (FDA) as a treatment option for NSCLC with *EGFR* mutations. In addition, afatinib is effective for managing brain metastasis or LMC in NSCLC who failed to respond to erlotinib or gefitinib [56]. While the study that determined this did not distinguish between brain metastases and LMC, median time to afatinib failure for patients with CNS metastasis (metastases or LMC) was 3.6 months, which was similar to the effects observed in a matched group of patients without CNS metastasis [56]. In addition, 35% of observable patients showed cerebral response [56]. However, the efficacy of afatinib in LMC patients who failed high-dose *EGFR* TKI was not reported. A third-generation *EGFR* TKI osimertinib (AZD9291) showed efficacy in an in vivo LMC model [57] that was resistant to first- and second-generation *EGFR* TKI. In addition, osimertinib at 160 mg once daily demonstrates encouraging preliminary safety and activity in heavily pre-treated patients with LMC from NSCLC with *EGFR* mutations [58]. AZD3759, a potent, CNS-penetrant *EGFR* TKI, achieved sufficient CNS exposure and demonstrated promising anti-tumor activity in a phase I study [59]. However, evidence is minimal for second- and third-generation *EGFR* TKI agents, so future studies are needed to confirm the efficacy of these agents in NSCLC with LMC.

### 4.2. Lung Cancer Patients with Anaplastic Lymphoma Kinase (ALK) Translocation

Rearrangement of the *ALK* gene is found in approximately 4%–5% of NSCLC cases and is more prevalent in nonsmokers, younger patients, and those with adenocarcinoma histology [60]. Detecting *ALK* rearrangement is important because *ALK*-positive NSCLC is highly sensitive to *ALK* inhibitors [61]. However, crizotinib, a first-generation *ALK* inhibitor, poorly penetrates the BBB, so the CNS remains a frequent site of relapse for *ALK*-positive patients treated with crizotinib [62]. In a single-case report, the CSF-to-plasma ratio of crizotinib was only 0.026 [63]. Ceritinib is a second-generation *ALK* inhibitor that is 20 times as potent as crizotinib, and is effective for *ALK* positive patients who have progressed while on crizotinib [64]. In another single case, ceritinib showed efficacy against LMC in ALK-positive patients who were resistant to crizotinib (Table 2) [65]. Alectinib is another second-generation *ALK* inhibitor that is active against crizotinib-resistant disease and in patients with brain metastases [66,67,68]. Alectinib also showed clinical activity against LMC [69,70]. In a study performed in a knockout mouse model, crizotinib and ceritinib were good transport substrates of human P-glycoprotein (ATP Binding Cassette Subfamily B Member 1, ABCB1), which means crizotinib and ceritinib were efficiently transported out of the brain by ABCB1 [71,72]. In contrast, alectinib was not a good ABCB1 substrate and showed a high brain-to-plasma ratio of 0.63–0.94 in a mouse model [73]. While prospective data are lacking, evidence from case reports and preclinical studies show that second-generation *ALK* inhibitors, especially ceritinib, may be treatment options in *ALK-*positive NSCLC with LMC.

### 4.3. Breast Cancer Patients with Human Epidermal Growth Factor Receptor 2 (HER2) Amplification

Amplification of *HER2* is observed in approximately 15%–20% of breast cancer patients [74,75]. Patients with *HER2* amplification have a more aggressive form of the disease, but the introduction of *HER2*-targeted therapy has improved the prognosis of *HER2*-positive patients [74,76]. While brain metastasis occurs more frequently in *HER2*-positive breast cancer, *HER2* status is not associated with an increased risk of developing LMC [77]. Because patients with *HER2-*positive breast cancer liver longer due to improvement in *HER2*-directed therapies, the incidence of LMC may rise.

Although trastuzumab, a monoclonal antibody that interferes with the *HER2* receptor, shows efficacy in *HER2*-positive breast cancer, its role in LMC is limited due to its large molecular size of 185 kDa [78]. Despite a possible increase in BBB permeability in brain metastases patients, CSF levels of trastuzumab were 300-fold lower than serum levels in breast cancer patients with brain metastases [78]. In a study by Stemmler et al. [79], the serum to CSF trastuzumab ratio in breast cancer patients with brain metastases was 420:1 prior to radiotherapy, 76:1 after radiotherapy, and 49:1 after radiotherapy in patients with concomitant LMC. Because systemic trastuzumab cannot readily cross the BBB, intrathecal trastuzumab has been attempted in cases of *HER2*-positive breast cancer patients with LMC. Even though there are no phase II/III clinical studies, intrathecal trastuzumab is effective at managing LMC as a single agent or in combination with other agents (intrathecal chemotherapy agents, systemic chemotherapy, or systemic anti-*HER2* therapy) [80,81,82,83,84]. A systemic review and pooled analysis of 13 articles (including 17 patients) revealed that intrathecal trastuzumab is a safe and effective option for *HER2*-postive breast cancer patients with LMC [77]. Intrathecal trastuzumab showed a tolerable safety profile across a wide dose range (single doses of 4–150 mg and total doses of 35–1100 mg) [77]. In a phase I study, intrathecal trastuzumab was given twice a week for 4 weeks, then once a week for 4 weeks, and then every other week until progression of the disease. Under this regimen intrathecal trastuzumab was well tolerated up to 80 mg [85]. Although, there is no standard dose, regimen, or schedule for intrathecal trastuzumab, this agent may be an option of *HER2*-positive breast cancer patients with LMC.

Lapatinib is a dual TKI of *HER1* and *HER2* that shows efficacy against metastatic *HER2*-positive breast cancer that has progressed after trastuzumab treatment [86,87]. In a single-arm phase 2 study, lapatinib plus capecitabine was active in managing brain metastases in patients with *HER2*-positive breast cancer who had not received previous whole brain radiation therapy [88]. However, in a phase III open-label study of lapatinib plus capecitabine versus trastuzumab plus capecitabine, there was no difference in the incidence of CNS metastases between lapatinib-capecitabine and trastuzumab-capecitabine [89]. In addition, there are no reports on lapatinib for treatment of LMC. Currently, there is no evidence of using lapatinib, trastuzumab emtansine, or pertuzumab for managing LMC in patients who have *HER2*-positive breast cancer. To date, intrathecal trastuzumab is the only targeted treatment that shows efficacy above conventional therapies for managing LMC in *HER2*-positive breast cancer patients.

### 4.4. CD20 Positive Lymphoma Patients and BRAF Mutated Melanoma Patients

Rituximab is an anti-CD20 monoclonal antibody that is effective for treating diffuse large-B-cell lymphoma (DLBL) [90]. However, because of its large size, when administrated in systemic therapy rituximab CSF levels are only 0.1% of serum levels, and systemic rituximab therapy did not reduce the risk of secondary CNS occurrence in patients with DLBL [91,92]. Poor CNS penetration has led clinicians to conduct a study using intrathecal rituximab. A phase I study of the intraventricular administration of rituximab in recurrent CNS and intraocular lymphoma has been conducted [93]. Intraventricular rituximab was administered once during the first week and twice per week thereafter for 4 weeks. Intraventricular rituximab was well tolerated in doses up to 25 mg, but dose-limiting toxicity (grade 3 hypertension) was experienced in two patients treated at the 50 mg dose level. The estimated distribution half-life and elimination half-life of a 25 mg dose of intraventricular rituximab were 3.0 and 34.9 h, respectively. Among 10 patients, meningeal response was detected in six patients, and one patient exhibited resolution of brain parenchymal lymphoma [93]. In another case-series report, intraventricular rituximab showed efficacy in six patients with relapsed CNS lymphoma [94]. Intraventricular injections of 10–40 mg rituximab yielded a total clearance of malignant cells in CSF for three patients and leptomeningeal lymphoma nodules disappeared in another patient [94]. These results illustrate the feasibility of intrathecal rituximab for cases of LMC with CD20-positive Lymphoma.

*BRAF* inhibitors such as vemurafenib and dabrafenib shows promising effect in *BRAF* mutated advanced melanoma patients. In a single patient case report, vemurafenib resulted in a significant clinical and imaging response as well as prolonged survival [95]. However, in a report from six melanoma patients, vemurafenib showed low brain-to-plasma ratio of 0.98% [96]. More evidences are needed to confirm the role of *BRAF* inhibitors in *BRAF* mutated melanoma patients with LMC.

## 5. Conclusions and Future Directions

LMC is a devastating disease that occurs in 1%–5% of patients with solid tumors. Conventional therapies including intrathecal chemotherapy, systemic chemotherapy, radiation therapy and surgery have been tested, but the prognosis remains very poor for LMC with a median overall survival of <3 months. Recently, molecular targeted agents have been applied to LMC and have shown groundbreaking results. There are also results indicating the use of molecular targeting agents through proper dose and administration are effective for treating LMC in selected patient subgroups (*EGFR* TKI in *EGFR*-mutated NSCLC, intrathecal trastuzumab in *HER2* positive breast cancer, and intrathecal rituximab in CD20 positive lymphoma). In addition, second- and third-generation *ALK* inhibitors could play a role in managing LMC in *ALK*-positive NSCLC patients. However, randomized clinical trial data is needed and the use of new molecular targeting agents requires further investigation. Nevertheless, because there are few options for treating LMC, and these agents show promising results, molecular targeting agents could be a novel therapeutic option for treating LMC in appropriate patient groups.

## Figures and Tables

**Table 1:** Epidermal growth factor receptor (*EGFR*) TKI in *EGFR* (+) non-small cell lung cancer (NSCLC) with leptomeningeal carcinomatosis.

References	Design	*N*	Targeted Agent	Result
Liao et al., 2015 [48]	Retrospective	75	Any *EGFR* TKI	*EGFR* TKI was effective in managing LMC of *EGFR* (+) patients.
Jackman et al., 2015 [50]	Phase I study	7	High-dose gefitinib	High-dose gefitinib was well tolerated and showed moderate CSF penetration.
Kawamura et al., 2015 [51]	Retrospective	35	High-dose erlotinib (*N* = 12) or Standard-dose *EGFR* TKI (*N* = 23)	High-dose erlotinib showed efficacy in patients with LMC.
Lee et al., 2013 [52]	Retrospective	25	Gefitinib (*N* = 11) or Erlotinib (*N* = 14)	Erlotinib had better control rate for LMC compared to gefitinib.
Hoggknecht et al., 2015 [53]	Retrospective	100 (LMC or brain metastasis)	Afatinib	Afatinib showed clinical effect in patients with CNS metastasis (brain metastasis or LMC).
Nanjo et al., 2015 [54]	In vivo imaging	LMC mouse	Third generation TKI (AZD9291)	AZD9291 showed response in LMC mouse models refractory to EGFR TKI.

Abbreviations: EGFR, epidermal growth factor receptor; TKI, tyrosine kinase; LMC, leptomeningeal carcinomatosis; CNS, central nervous system.

**Table 2:** Anaplastic lymphoma kinase (*ALK*) inhibitor in *ALK* (+) NSCLC with leptomeningeal carcinomatosis.

References	Design	*N*	Targeted Agent	Result
Arrondeau et al., 2014 [60]	Case report	1	Ceritinib	Certinib showed clinical and radiographic improvement in LMC for over 5.5 months.
Ou et al., 2015 [64]	Case report	1	Alectinib	Alectinib induced durable (>15 months) complete response of LMC.
Gainor et al., 2015 [65]	Case series	4	Alectinib	3 (75%) experienced clinical and radiographic improvement in LMC. Another one patients had stable intracranial disease for 4 months.

Abbreviations: ALK, anaplastic lymphoma kinase; TKI, tyrosine kinase; LMC, leptomeningeal carcinomatosis.
